# How Does Gender Affect Sustainable Intensification of Cereal Production in the West African Sahel? Evidence from Burkina Faso

**DOI:** 10.1016/j.worlddev.2016.12.003

**Published:** 2017-04

**Authors:** Veronique Theriault, Melinda Smale, Hamza Haider

**Affiliations:** Michigan State University, East Lansing, USA

**Keywords:** adoption, cereals, gender, intensification, Burkina Faso, West Africa

## Abstract

Better understanding of gender differences in the adoption of agricultural intensification strategies is crucial for designing effective policies to close the gender gap while sustainably enhancing farm productivity. We examine gender differences in adoption rates, likelihood and determinants of adopting strategy sets that enhance yields, protect crops, and restore soils in the West African Sahel, based on analysis of cereal production in Burkina Faso. Applying a multivariate probit model to a nationally representative household panel, we exploit the individual plot as unit of analysis and control for plot manager characteristics along with other covariates. Reflecting the socio-cultural context of farming combined with the economic attributes of inputs, we find that female managers of individual cereal fields are less likely than their male counterparts to adopt yield-enhancing and soil-restoring strategies, although no differential is apparent for yield-protecting strategies. More broadly, gender-disaggregated regressions demonstrate that adoption determinants differ by gender. Plot manager characteristics, including age, marital status, and access to credit or extension services do influence adoption decisions. Furthermore, household resources influence the probability of adopting intensification strategy sets differently by gender of the plot manager. Variables expressing the availability of household labor strongly influence the adoption of soil-restoring strategies by female plot managers. By contrast, household resources such as extent of livestock owned, value of non-farm income, and area planted to cotton affect the adoption choices of male plot managers. Rectifying the male bias in extension services along with improving access to credit, income, and equipment to female plot managers could contribute to sustainable agricultural intensification.

## Highlights

• We examine gender differentials in technology adoption on individually managed cereal plots.
• Female plot managers are less likely to adopt yield-enhancing and soil-restoring strategy sets than male plot managers.
• The socio-cultural context and economic attributes of the technology set affect incentives to adopt.
• We demonstrate that plot manager characteristics do influence adoption decisions.
• Household resources affect male and female plot managers differently.

## 1 Introduction

With uncertain rainfall and degraded soils, farmers in the West African Sahel have devised techniques for managing their land and water intensively in order to feed their families (e.g., Reij, Tappan, & Smale, 2009). Donors and governments have also invested in agricultural research to raise productivity since the great droughts of the 1970s and 1980s. Nonetheless, farm families in this region remain vulnerable to chronic food insecurity. Increasing food supply on smallholder farms will depend on sustainable agricultural intensification. Given the key role that women play in food security on smallholder farms (e.g., Quisumbing *et al*., 2014), better understanding of gender differences in the adoption of intensification strategies is crucial for designing effective policies to close the gender gap while sustainably enhancing farm productivity.

Developing agricultural technologies to improve women’s well-being poses a “much more difficult challenge” than merely “taking women into account” (Doss, 2001, p. 2057). Indeed, gender roles in farming and farm household structures vary widely across cultural contexts. In the West African Sahel, extended farm households headed by a patriarch continue to prevail (Guirkinger and Platteau, 2014, West, 2010). Extended farm households are composed of members who are vertically (e.g., married sons and their wives) and horizontally (e.g., brothers and multiple wives) linked to the household head, who is responsible for managing collectively farmed plots and allocating individually managed plots among household members.

In conducting gender research, the selection of the appropriate unit of analysis matters. A large body of case studies conducted in Africa controls for the sex of the household head when estimating the determinants of technology adoption and productivity. In their seminal work, Doss and Morris (2001) found no gender difference in the adoption of improved seed and fertilizer on maize in Ghana, but major differences when they accounted for the sex of the household head; females in female-headed households were systematically disadvantaged and adopted less frequently than those in male-headed households. Subsequent analyses have shown that it is not whether the head is female but rather the lack of access to resources that explains gender productivity differentials (e.g., Alene et al., 2008, Quisumbing and Pandolfelli, 2010).

Similarly, Peterman, Quisumbing, Behrman, and Nkonya (2011) demonstrated the sensitivity of productivity differentials to whether comparisons were made at the level of the household head or between male and female plot managers using datasets from Nigeria and Uganda. In Burkina Faso, Udry (1996) found productivity differentials between plots managed by men and women. With the same dataset and a comparable modeling framework, Kazianga and Wahhaj (2013) later attributed the differential to headship management of collective fields, finding no differential between plots managed individually by males and females. In neither of these studies, however, were researchers able to control for whether individual plots were managed by the household head.

Several recent studies have examined the determinants of adoption of multiple yield-enhancing, protective, and/or conserving strategies, especially in Eastern and Southern Africa (Kamau et al., 2014, Kassie et al., 2013, Lambrecht et al., 2016, Ndiritu et al., 2014, Teklewold et al., 2013, Wainaina et al., 2016), and to a lesser extent in the West African Sahel (Asfaw, Di Battista, & Lipper, 2016). Although most of those studies have controlled for gender of the farmer or household head, better understanding of gender differences in technology adoption has not been their main focus, with the exception of Ndiritu *et al*. (2014)’s work in Kenya and the study by Lambrecht *et al*. (2016) in DR Congo.

Ndiritu *et al*. (2014) found that female plot managers were less likely to adopt minimum tillage and use manure, suggesting that socioeconomic inequalities and barriers in access to resources still exist for women. The authors underscored the need to conduct more analysis on the adoption of multiple intensification strategies at the individual decision-making level, but no plot manager characteristic other than the sex of the plot manager was included in their analysis. The effect of participation in agricultural extension programs by male and female farmers on technology adoption was at the core of the study by Lambrecht *et al*. (2016), who found that joint male and female participation led to highest adoption rates of improved seed, fertilizer, and row planting.

In this analysis, we examine three fundamental questions regarding gender and technology adoption in cereal production. We apply multivariate probit models to test for gender differentials in (1) adoption rates; (2) the likelihood of adoption; and (3) determinants of adoption in the farming context of the West African Sahel, using Burkina Faso as an example. We consider the adoption of yield-enhancing, yield-protecting, and soil restorative strategy sets on individually managed plots of maize, sorghum, and millet.

Our analysis contributes to the literature on gender roles and technology adoption in several ways. First, we add to the ongoing discussion on the gender gap in technology adoption by exploring jointly the determinants of a wide range of intensification strategy sets in a different farming context- the West African Sahel. Second, reflecting the cultural specificity of gender roles and farm decision-making in this region, we control not only for the sex of the plot manager but whether or not the manager is the household head. Third, we use a nationally representative household panel to exploit the individual plot as a unit of analysis and control for additional plot manager characteristics along with other covariates.

## 2 Gender and farm structure in the West African Sahel

Historical, ethnographic research (e.g., Hammond, 1966, Lallemand, 1977) and more recent research by Udry, 1996, West, 2010, and Kazianga and Wahhaj (2013) notes the defining features of Burkinabe farm households, also depicted by Van den Broek (2009) and Guirkinger and Platteau (2014) for cereals production systems in Mali. Farm work is conducted across numerous plots with well-defined boundaries, for which the management has been assigned to a specific adult member, under the titular authority of the household head who is almost always an elder patriarch. Some plots are worked collectively by household members. According to existing social norms, these are managed by the household head and all proceeds are destined to meet the needs of the extended family. Other plots are managed by individual male or female members who make decisions over input use, including the choice of crops and techniques. Proceeds from production on individual plots are retained by the household member to meet his or her food or budgetary needs. This individualization of decision-making power differs from parts of Eastern and Southern Africa, where jointly managed plots are common (see Ndiritu *et al*., 2014 for Kenya; Marenya, Kassie, & Tostao, 2015 for Mozambique; Slavchevska, 2015 for Tanzania).

The allocation of individual plots is associated with the status of household members in relation to the patriarch (Kazianga and Wahhaj, 2013, Kevane and Gray, 1999). For example, married sons of the patriarch and some unmarried sons are often allocated their own fields. Upon marriage to sons of the patriarch, wives are allocated small plots to meet the specific needs of their own children and to contribute ingredients used in preparation of meals. In some instances, an elderly widow of a male family member may be allocated fields to ensure her subsistence. Even when land has been allocated, the extent to which major factors of production such as labor and draught power are shared is the outcome of a complex intrahousehold negotiation (De Vreyer et al., 2008, Kevane and Gray, 1999, Smith and Chavas, 2007). Ethnographic studies (Becker, 1990, Becker, 1996, Kevane and Wydick, 2001) have long reported that, as a reflection of the priority placed on overall household welfare, household labor is allocated first to the collective fields. Household members can work on their individual fields after completing all their tasks on the collective fields.

Another important feature of the Burkinabe farming structure is the interplay between customary norms and formal tenure rights. Konaté (2006) explains that in patrilineal systems such as that of Burkina Faso, land rights are transmitted via male family members. Because of the principle of exogamy (marriage outside the family), women are generally awarded no more than usufruct rights at marriage. The possibility of divorce, and thus alienation of lineage lands outside the family, poses an inherent threat. Thus, despite that all Burkinabe are equal in the rights according to the Constitution, and that the Agrarian Reform of 1996 declares no discrimination, customary norms, which are inherently unequal, prevail in practice. For instance, in some regions of Burkina Faso, women have no access to plots except for the off-season (Konaté, 2006). Another example of gender inequality is the allocation of irrigated plots to male farmers only in Dakiri, eastern Burkina Faso (Zwarteveen, 1997).

In recent years, socio-cultural norms and incentives guiding resource use have evolved in response to food insecurity, spurred in part by the land fragmentation that accompanies high rates of population growth (Kazianga & Wahhaj, 2013). Guirkinger and Platteau (2014) document the individualization of agricultural production within extended farm households, as more plots are allocated by the patriarch to both male and female household members. Micro-studies also suggest that more women are planting sorghum—a crop traditionally cultivated on the collective fields and managed by the elder—on their individual plots, in order to contribute to family food security, and also because sorghum prices have risen relative to other crops they grew before (Dabiré et al., 2016, Van den Broek, 2009). Hence the importance of examining the determinants of adoption of multiple intensification strategies for cereal plots that are individually managed by men or women.

## 3 Data

The data for this analysis are drawn from the Continuous Farm Household Survey (*Enquête Permanente Agricole* (EPA)) collected by the General Research and Sectoral Statistics Department (*Direction Générale des Études et des Statistiques Sectorielles* (DGESS)) of the Ministry of Agriculture and Food Security (*Ministère de l’Agriculture et de la Sécurité Alimentaire* (MASA)) in Burkina Faso. The sampling frame for the EPA is based on the 2006 Population Census. The nationally representative sample includes 4,130 household farms in 826 villages across all 45 provinces. The EPA generates production, area and yield data for rainfed crops, serves as an early warning system for food insecurity, and also furnishes general information about livestock holdings, income and expenditures of rural households, and farm input use. In this article, we utilize data covering the 3-year period from 2009–10 through 2011–12, which are the last years for which fully cleaned data are available. The format of the EPA data reflects the socio-demographic structure of farm family decision-making in Burkina Faso, as described in Section 2. The enumerator instructions state that all questions regarding inputs and outputs on individual plots were answered by plot managers.

After excluding households that were not continuously surveyed throughout the three year period and those that did not grow cereal crops, we are left with over 2,700 households cultivating about 11,000 cereal plots, including roughly 3,000 individually managed cereal plots each year. Cereal plots refer to those where the primary crop is sorghum, millet or maize. The data constitute a panel at the household level but not by plot. Although the same households were surveyed during the three year period, it is important to note that plot *j* in 2009–10 in household *i* may be different from plot *j* in 2010–11 and/or 2011–12. In order to conduct the analysis at the plot level, we pool all individually managed cereal plots into a large single dataset of 9,659 observations.

We draw rainfall estimates from the National Oceanic and Atmospheric Administration’s Climate Prediction Center at the commune level to measure rainfall variability. The source of data on population density is the National Institute of Statistics and Demography (*Institut National de la Statistique et de la Démographie*) (INSD, 2014).

## 4 Empirical strategy

We utilize a straightforward latent variable framework to model the decision of individuals (male and female plot managers) to adopt as a binary outcome. Consistent with the overall perspective of decision-making within a complex household in the face of imperfect or missing markets, we consider that individual choices are conditioned on household and market-related characteristics. Recognizing that even if markets were perfect and complete, differentiation in input use could result from plot-specific variable features of land and micro-climate, we also model choices as conditional on plot and agro-climatic characteristics. As explained in the introduction, consistent with current analyses of gender-differentiated adoption and productivity, we include plot manager characteristics other than gender as key covariates for hypothesis testing.

It is expected that individual family members will adopt intensification strategy sets that maximize their perceived benefits. Let the perceived benefits derived from adopting an intensification strategy set by farmer *i* at time *t*, be:

(1) $$y_{\mathrm{it}}^{*}=X_{\mathrm{it}}\beta +u_{\mathrm{it}}+c_{i}\;i=1\text{,}\ldots n\text{,and}\;t=1\text{,}\ldots \text{T}$$

where, *X_it_* is a set of observable covariates, *β* is a vector of parameter estimates, *u_it_* is the normally distributed error term independent of *X_it_*, and *c_i_* are the time-invariant unobserved effects (Greene, 2003, Hsiao, 2003). Although, perceived benefits derived from a strategy set are unobservable ($y_{\mathrm{it}}^{*}$), the decision to adopt is observable (*y_it_*) and is expressed as:

(2) $$\begin{array}{cc} y_{\mathrm{it}} & =1\;\text{if}\;y_{\mathrm{it}}^{*}>0\\ & =0\;\mathrm{otherwise} \end{array}$$

Here, *y_it_* is a limited dependent variable, which takes a value of 1 if the farmer *i* makes the observable decision of adopting any intensification strategy within a set at time *t* and zero otherwise. As specified, both *c_i_* and *β* are unknown parameters to be estimated by the probability model (Greene, 2010, Hsiao, 2003):

(3) $$\text{Prob}(y_{\mathrm{it}}=1|X_{\mathrm{it}}\text{,}c_{i})=F(X_{\mathrm{it}}\beta +c_{i})$$

Given the binary nature of the dependent variable (to adopt or not), the possible interdependence across a set of intensification strategies, a multivariate probit model is used to examine the gender differentials in the determinants of adoption.

The multivariate probit model can be seen as an extension of the probit model, since it allows estimating several probit models simultaneously, while allowing the error terms in those models to be correlated (Greene, 2003). Ignoring the correlation across error terms would lead to inefficient coefficient estimates and thereby erroneous inference (Hsiao, 2003). Correlation occurs when unobservable characteristics (e.g., intrinsic management skills) captured in the error terms influence the adoption decision of intensification strategies. Significantly positive correlations in the error terms have been interpreted as evidence of complementarity between strategies, whereas significantly negative correlations have been interpreted as evidence of substitutability (Asfaw et al., 2016, Dorfman, 1996, Khanna, 2001, Ndiritu et al., 2014). This relies, however, on the assumption that unobserved heterogeneity is uncorrelated with explanatory variables. We exploit the panel nature of the data to control for time-invariant household heterogeneity, and control for a large number of plot manager and plot characteristics that affect adoption of these practices.

In nonlinear models estimated with data that have a time dimension, the use of a fixed-effect approach to control for unobserved, time-invariant effects is problematic, since it leads to inconsistent parameter estimates (Greene, 2010, Hsiao, 2003). This is known as the incidental parameter problem, which also occurs with the inclusion of household-level dummy variables (Wooldridge, 2015). A commonly used approach to address this problem in panel data estimation is Correlated Random Effects (CRE), also known as the Chamberlain–Mundlak device (Cameron and Trivedi, 2005, Chamberlain, 1984, Mundlak, 1978, Wooldridge, 2010). The CRE approach allows for unobserved time-invariant effects or unobserved heterogeneity (*c_i_*) to be correlated with observed covariates in nonlinear models, through the projection of those effects on the time average (${\overset{¯}{X}}_{i})$of covariates:

(4) $$c_{i}={\overset{¯}{X}}_{i}\delta +\alpha_{i}+\omega \text{,}\;\alpha_{i}|X_{i}∼N\left(0\text{,}\sigma_{\alpha }^{2}\right)$$

Following this approach, we estimate the reduced form

(5) $$\text{Prob}(y_{\mathrm{it}}=1|X_{\mathrm{it}})=\text{F}(X_{\mathrm{it}}\beta +{\overset{¯}{X}}_{i}\delta +\alpha_{i}+\omega )\text{,}$$

where ${\overset{¯}{X}}_{i}$ are the means of covariates that vary over time for household *i,* is the normally distributed error term, and other parameters are as defined above. Wooldridge (2015) refers to the class of models we estimate as the “unobserved effects” nonlinear models, in which the empirical interest is to estimate a response probability in a microeconomic setting, while also addressing the problem of heterogeneity (*c_i_*). According to Wooldridge (2015, p. 4), the focus of the nonlinear unobserved effects model is on “parametric approximations that are logically consistent” with the binary nature of the dependent variable rather than on estimation of parameters *per se*. Though a linear model is a useful starting point for estimating average partial effects, the model imposes the restriction that 0 ⩽ *X_it_β* + *c_i_* ⩽ 1 for all *X_it_*. The linear functional form for equation 5 cannot hold over a wide range of values of *X_it_* and *c_i_*, and may thus may provide a poor approximation (Wooldridge, 2015).

Eqn. (5) is the basis of the econometric estimation. Since the same households (and not plots) are tracked over time, the CRE approach is applied at the household level. Plot characteristics are included as regressors to capture differences across plots. All regressions are estimated with time effects (year dummy variables) to allow for time trends. Standard errors are clustered at the household level to reflect that decisions made by household members may be mutually influenced and allow for correlation among the unobservable characteristics of plot managers belonging to the same household.

## 5 Variables

The yield-enhancing, yield-protecting, and soil restorative strategy sets differ in terms of their economic attributes, which in turn affects adoption incentives. The yield-enhancing set is composed of inorganic fertilizer and improved seeds. Conventionally, since the Green Revolution, these have been promoted by extension and credit services as fixed-size input packages that have been tested on-station to optimize complementarities in yield potential (Feder, Just, & Zilberman, 1985). Farmers choose to use them at the outset of the rainy season, based on expected yield. By contrast, farmers decide to adopt inputs such as herbicide, fungicide and pesticide in response to perceived threats or observed damage from plant predators or disease (Lichtenberg & Zilberman, 1986). Comparing these two sets, fertilizer (and the input package) is viewed as a “lumpy” input due to its weight, with high costs of transport, especially in land-locked countries—making it non-neutral to scale. Damage control inputs are highly divisible, or neutral to scale.

The soil restorative strategy set includes annual practices aimed at controlling the moisture environment of the plant, reducing erosion and water run-off (planting pits), and organic fertilizer, which is recommended in combination with these to restore soil fertility, in terms of its physical, chemical, and biological properties (GIZ, 2012, Reij et al., 2009). In contrast to the other two sets, the soil restorative strategy set demands considerable labor investment but also requires access to equipment for manure transport. Moreover, the effects of soil restorative practices have a longer time horizon. Finally, damage control inputs can be substituted directly by use of labor, such as weeding. Labor cannot substitute directly for seed, fertilizer, manure, or restorative soil structures.

Table 1 provides detailed definitions of each intensification strategy set along with summary statistics. Overall, adoption of agricultural technologies is low, with use of at least one intensification strategy on only 40% of individual plots in the sample.

The choice of explanatory variables draws from the concepts we outline above and a vast theoretical and empirical literature on the adoption of agricultural innovations in developing country agriculture, including reviews by Feder *et al*. (1985), Feder and Umali (1993), and more recently by Foster and Rosenzweig (2010). A principle in this literature is that costs, and access to information about a new technology, are related to capital endowments, such as farm size. In their overview, Sunding and Zilberman (2001) emphasize that adoption of technology often occurs in response to constraints and/or to seize economic opportunities. There is a wide consensus in the literature regarding the factors that influence agricultural technology adoption. Among these factors, financial and labor constraints along with land tenure have often been found to influence farmers’ adoption decision in regard to intensification strategies (examples of recent studies include Arslan et al., 2014, Kamau et al., 2014, Kassie et al., 2013, Kassie et al., 2015, Ndiritu et al., 2014, Teklewold et al., 2013). After reviewing many empirical studies on soil conserving practices, Knowler and Bradshaw (2007) were unable to identify factors that uniformly explain adoption across crops, regions, and technology. These and other authors (e.g., Tittonell, Vanlauwe, Leffelaar, Rowe, & Giller, 2005) highlight the context-specificity of the combinations of practices that farmers find optimal under their conditions.

Table 2 provides definitions of the explanatory variables used in our econometric models and summary statistics. We group factors by plot manager, plot, household, market, and agro-climatic characteristics. Summary statistics disaggregated by gender are available in Appendix (see Table 6).

In addition to gender, plot manager characteristics include age, education, marital status, and headship, as well as plot manager access to credit and extension services. The number of years since plot managers received any extension services is expected to be negatively correlated with technology adoption. Credit access is defined as whether or not the plot manager reported access to credit in the preceding year, regardless of whether credit was used in the survey season. Plot physical characteristics include size, location, and topography (i.e., lowland, slope).

We then include the covariates known to explain differences in input adoption among households, including wealth, tenure security, and availability of labor, which is a complementary input for all intensification strategy sets. Total landholding, livestock, non-farm income, and number of cotton hectares are used as proxies for wealth. The number of livestock owned also provides information regarding the availability of manure. Households growing cotton benefit not only from the cash it generates, but from better access to inputs on credit and extension services provided through the cotton ginneries (Theriault & Tschirley, 2014). We expect decisions regarding the scale of cotton production to precede or predetermine the choice of technology on cereal plots. Labor availability is measured as the total number of male and female adults in the household and as the overall ratio of children to women. The tenure variable measures the share of plots that have secure rights over the total number of plots within a household. The time-averages of landholding, non-farm income, and cotton variables, which vary over survey years, are included in the models to control for correlated random effects.

Market-related factors affect adoption incentives via transaction costs and endogenous prices. The share of households in the village who are members of a cereal association is included to express access to socio-political capital as exogenous to the decisions of individual plot managers. Variables measuring the number of agro-dealers and population density at the province level are included as proxies for market access.

To account for potential interannual fluctuation in moisture availability for cereal crops, the coefficients of variation in total annual rainfall at the commune level over the last three years are included. Cereal dummy variables are also included to control for crop differences.

## 6 Results

### (a) Gender differentials in adoption rates

Table 1 reports adoption rates on individually managed cereal plots for each of the intensification strategy set, including all plots and differentiating by gender of the plot manager. Whether female managers adopt intensification strategy sets at lower rates than their male counterpart is tested with a simple comparison of means, without controlling for other covariates. Differences in adoption rates between men and women are statistically significant for all three intensification strategy sets, as shown by the Pearson Chi-squared tests. The adoption rate for the yield-enhancing set is 10% on average, but with men adopting twice as much than women. The yield-protecting set is adopted on 17% of individually managed cereal plots, but differences between men and women plot managers, although statistically significant, are not likely to be meaningful. Overall, the soil-restoring set is the most adopted (25%) across all plot managers, but adoption rates remain higher for men compared to women.

### (b) Gender differentials in the likelihood of adoption

Table 3 shows the results from the multivariate probit (MVP) regression for all cereal individual plots. The likelihood ratio test leads us to reject the null hypothesis of independent error terms overall and across intensification strategy sets (*p*-value 0.0000). Multivariate probit is preferred statistically to univariate probit regressions, indicating that the probability of adopting one set is interdependent on the decision of whether to adopt another set. The estimates of the correlation between the regression error terms are positive and significant, suggesting complementarities across the yield-enhancing, yield-protecting, and soil-restoring sets.

The regression coefficients in Table 3 can be interpreted in terms of sign but not as marginal effects.^1^ For illustrative purpose, marginal effects derived from univariate probit models, which present some efficiency losses compared to the multivariate probit model, are presented in Appendix (see Table 7). The results from the univariate probit models suggest that being female reduces the probability of adopting the yield-enhancing and soil-restoring sets by 2 and 6 percentage points. Those effects are large in magnitude, given that the average rates of adoption of the yield-enhancing and soil-restoring sets are about 10% and 25% respectively.

Female plot managers are significantly less likely to adopt either the yield-enhancing or the soil-restoring strategy sets, after controlling for other covariates. This finding is consistent with the notion that within households, women have less access to these inputs. Fertilizer, which is lumpy and costly per unit, is often extended via formal programs and projects to the household head. To utilize soil restorative practices, plot managers need access to larger livestock, additional labor and equipment, which are typically managed by the head on behalf of the household since they are deployed across numerous plots and crops. By contrast, no gender differential is evident in the probability of adopting yield-protecting (damage control) inputs, which are divisible, scale-neutral, and widely available through agro-dealers (Haggblade, Smale, Kergna, Theriault, & Assima, 2016).

Our findings are consistent with some previous studies regarding gender differences in fertilizer use (Gilbert et al., 2002, Udry, 1996). Ndiritu *et al*. (2014) found no gender difference in the adoption of fertilizer but did find them for use of manure and minimum tillage. This last finding, and ours, does not mean that the gender of the farmer influences adoption decision *per se* but rather reflects how the socio-cultural farming context combined with the economic attributes of technology affect adoption. We examine this in greater detail below, when testing for underlying differences in the determinants of adoption by men and women.

### (c) Gender differentials in the determinants of adoption

To test whether the determinants of adoption differ by gender of plot manager, we compare the (restricted) regression for all plot managers to separate (unrestricted) regressions for males and females only using a modified Chow test that is appropriate for nonlinear regressions (Greene, 2003). Comparing the regression for all plot managers to the separate regressions, we reject the hypothesis that regression parameters are the same for male and female plot managers (the log-likelihood ratio of 456 *vs*. Chi-squared critical value of 112 with 75 d.o.f at 1% significance). In other words, the underlying process that explains adoption differs between male and female plot managers. The statistical results are supported by observable differences in statistically significant determinants between the two sets of regressions (Table 4, Table 5). While some factors are shared, several are specific to either male or female plot managers.

Among the socio-demographic characteristics, age of the plot manager positively influences the probability of adopting the soil-restoring strategy set for both males and females. In this society, where elderly people are highly regarded (West, 2009), the status of older women within the household nearly approach that of men (Udvardy & Cattell, 1992). Both older male and female plot managers can use their authority to gain access to the household resources needed to adopt the soil-restoring strategy set. Being the head of the household does not statistically influence adoption of the intensification strategy set on individual plots, regardless of the gender of the plot manager. The marital status of the plot manager influences the probability of adopting the yield-enhancing set for both males and females but only the soil-restoring set for males. Since, in addition to age, marital status is highly valued in this society, younger unmarried males and females have a lower social status along with widows (Van de Walle, 2013, West, 2009). Moreover, married men can more easily adopt labor-intensive intensification strategies, such as the soil-restoring set, because they have greater rights over their wives’ labor allocation than married women have over the labor of their husbands or sons (Becker, 1990, Udvardy and Cattell, 1992, Van de Walle, 2013).

Extension services, which mostly target male farmers, statistically influence their decision to adopt modern inputs. The longer it has been since male plot managers received any extension services, the less likely they are to adopt the yield-enhancing and yield-protecting sets. This finding adds to the evidence that receiving advice from extension agents positively influences adoption of yield-enhancing inputs (Lambrecht et al., 2016, Ragasa et al., 2013) and damage control inputs. Whether plot managers have had access to credit over the last twelve months influences their probability to adopt intensification strategy sets. Being credit-unconstrained positively affects the probability of adoption of both modern input sets by female plot managers. In comparison, male plot managers, who are more often credit-unconstrained, are more likely to adopt the yield-enhancing strategy set but less likely to adopt the soil-restoring set.

Plot characteristics, such as topography, size, and location from residence influence the adoption of intensification strategy sets. The probability of adopting the soil-restoring strategy set, for both female and male farmers, is lower on plots that are considered far from the residence, since distant plots require more time, labor, and energy in transport. The larger is the plot size, the higher is the probability of adopting modern inputs (yield-enhancing and yield-protecting sets) for both male and female plot managers. In contrast, the plot’s topography affects adoption of intensification strategy sets differently for male and female plot managers. Although men are as likely as women to manage plots on land with slopes, the probability of adopting yield-enhancing and soil-restoring sets on these plots is statistically significant for women only. On lowland farm plots, the yield-enhancing set is more likely to be adopted by women but less likely to be adopted by men.

The probability of adopting intensification strategy sets on individual cereal plots is influenced by household characteristics, but the influence of these factors depends on the gender of the plot manager. In a patrilineal society, such as in Burkina Faso, women have less bargaining power than men, which limits their access to and control over household resources (Kevane and Gray, 1999, Nikiema et al., 2008), which in turn, affects their incentives to make investments on their plots. This is reflected by the high significance of many household characteristics, landholding, livestock, and non-farm income for male plot managers only. Farmers growing cotton in rotation with cereals have traditionally benefited from better access to credit and subsidized prices on yield-enhancing inputs and even training on how to make organic fertilizer (Theriault & Tschirley, 2014). Our results suggest that the benefits from cotton, which remains a men’s crop (Bassett, 2010), have largely bypassed women. Indeed, total cotton hectares influence positively the yield-enhancing and soil-restoring sets for men only.

In contrast, factors related to labor availability more significantly affect the decision to adopt intensification strategy sets for female compared to male plot managers. These results reinforce the idea that women’s access to labor plays a crucial role in technology adoption (Quisumbing & Pandolfelli, 2010). Furthermore, female plot managers in households that have secure rights on a greater share of their plots are more likely to adopt yield-enhancing and soil-restoring strategy sets. Within farm households with greater tenure security, female plot managers may feel less threatened to lose their usufruct rights, and thereby, have more incentives to adopt intensification strategies with medium to longer term impacts, since they are more likely to reap the benefits from their investment. These results are consistent with previous studies (Kassie et al., 2013, Kassie et al., 2015, Ouédraogo et al., 2001).

Gender differences are apparent in the ways market factors affect the probability of adopting intensification strategy sets. Villages that have a large share of households belonging to a cereal association are more powerful in attracting resources and public support. By serving as platforms to share general knowledge and tips regarding agricultural practices, cereal associations positively influence technology adoption, especially for female plot managers. The number of agro-dealers in each province negatively influences adoption of the yield-enhancing and soil-restoring sets, but positively influences adoption of the yield-protecting set. This reflects the weaknesses of the Burkinabe private input supply sector, especially in regards to yield-enhancing inputs. The public sector has been highly involved in the marketing of fertilizer and improved seeds, notably in the cotton-cereal farming system, which has led to the displacement of private agro-dealers (Theriault & Tschirley, 2014). These yield-enhancing inputs are provided directly to cotton farmer associations, mostly composed of male famers, by-passing agro-dealers. As a consequence, more agro-dealers are moving their focus from fertilizer to horticultural inputs, which include herbicide, fungicide, and pesticide (Holtzman, Kabore, Tassembedo, & Adomayakpor, 2013). High population density negatively influences adoption of the yield-protecting set, which may suggest a preference for manual labor where there is no labor shortage (i.e., hand weeding instead of herbicide use; insect scooting instead of insecticide spraying).

The coefficient of variation of annual rainfall at the commune level is positive and significant for two out of three intensification sets, regardless of whether the plot manager is male or female. This suggests that all farmers respond to increased variability in water supply in their commune by adopting yield-enhancing and soil-restoring sets. Our results are consistent with Arslan *et al*. (2014) who found that longer delays on the onset of the rainy season and higher rainfall variability lead to higher level of adoption of conservation farming and minimum soil disturbance in Zambia.

The choice of crops also influences adoption decisions, in similar ways for both male and female plot managers. Compared to sorghum, maize plots are more likely to benefit from the adoption of yield-enhancement and soil-restoring strategy sets by both male and female managers. This finding reflects the economic importance of maize as a cash crop in this region. In contrast, the adoption of yield-protection strategies is less likely on millet than on sorghum plots for either male or female managers. Millet, a subsistence crop, is more widely grown in drier regions, where pest and disease pressures may be lower.

## 7 Conclusions and policy implications

In this article, we have tested gender differences in the rates, likelihood, and determinants of adoption of sustainable intensification strategies between male and female plot managers growing cereals in the West African Sahel. We contribute to the literature on the gender gap in technology adoption by controlling for the sex of the plot manager along with other plot manager characteristics (i.e., age, marital status, headship, education, access to credit, and extension services). We also contribute to the literature on adoption of sustainable farming practices by exploring the use of numerous inputs grouped in terms of strategies (yield-enhancing, yield-protecting, soil-restoring sets), and their interrelationships, while controlling for major cereal crops (sorghum, maize, millet).

Overall, the soil-restoring strategy set is the most frequently adopted, followed by the yield-protecting and the yield-enhancing, regardless of whether the plot manager is male or female. Findings from the descriptive statistics also suggest that female plot managers are adopting any of the three intensification strategy sets at lower rates.

More nuanced results emerge from the econometric analysis. The likelihood ratio test confirms the interrelatedness of intensification strategy sets and the appropriateness of the multivariate probit model as compared to independent probit models for predicting adoption. Results from the regression that includes a dummy for the sex of the plot manager show statistically significant differences in the probability of adopting the yield-enhancing and soil-restoring sets, after controlling for other plot manager characteristics and covariates. In contrast, sex of the plot manager does not statistically influence the probability of adopting the yield-protecting strategy set. This finding reflects how both the gendered, socio-cultural farming context and economic attributes of the technology affect incentives to adopt.

Disaggregated regressions demonstrate that the determinants of adoption differ between male and female plot managers. Plot manager characteristics, including age, marital status, and access to credit or extension services do influence adoption decisions, but in different ways according to the sex of the plot manager. Access to extension is significant for male managers, who have been targeted by programs in the past—but not for female managers. Furthermore, household resources influence the probability of adopting intensification strategy sets differently by sex of the plot manager. Variables expressing the availability of household labor strongly influence the adoption of soil-restoring strategies by female plot managers. By contrast, household resources such as extent of livestock owned, value of non-farm income, and area planted to cotton more often affect the adoption choices of male plot managers.

Our results provide insights on the influence of gender roles and economic attributes of technology on adoption decision that are relevant to the design and implementation of effective policies to sustainably increase production by farmers in Burkina Faso and similar areas of the West African Sahel. The interrelatedness of adoption strategies (yield-enhancing, yield-protecting, and soil-restoring sets) confirms the policy importance of designing extension mechanisms and messages that encourage farmers to consider flexible combinations of techniques and practices so that they can benefit from agronomic and economic complementarities. The promotion of any single intensification strategy should take incentives for use of other sets, and current patterns of input use, into consideration.

At the same time, the variation in input use confirms that farmers in these environments are not best approached with a fixed package in mind, but instead, with a recognition that due to constraints related to microclimate, labor, and cash, they will need the option of selecting from “menus” of practices and to suit their own conditions. Although plant breeders and agronomist must work to achieve complementarity by designing optimal packages of inputs, farmers’ optimal combinations will be as heterogeneous as the farm population and agro-ecology. The significance of plot characteristics in our models also supports this notion. Future research might explore the relative costs and benefits of research-and-extension designs that support this heterogeneity rather than blanket, uniform recommendations.

Gender differentials in adoption rates for yield-enhancing and soil-restoring sets and among determinants of adoption confirm the need to collect data disaggregated at the plot level and design and promote policies that, while respecting socio-cultural norms, also respect opportunities and incentives for individuals within multigenerational, multi-family farms.

The role of public extension services, for which funding support has declined in recent decades in favor of other types of information provision, nonetheless appears to be important in adoption decisions, especially in regards to modern inputs. Some changes are needed to redress the male bias in extension services, by including women as beneficiaries and covering topics on sustainable intensification, which takes into account women’s constraints to technology adoption. Credit access also remains crucial for inputs purchased with cash (yield-enhancing and yield-protecting sets), especially for women who have limited economic control within the household. Improving access to education, income, and equipment to women could contribute to increase their bargaining power and thereby, adoption of sustainable intensification strategies.

Customary norms also appear to play key roles in adoption of strategies for sustainable intensification of agriculture. Marriage, in particular, appears as significant for young men, as compared to women plot managers, most of whom obtained usufruct rights upon marriage. Among men, major differences in social status are related to headship and marriage. Only a tiny minority of women in the sample are heads. Looking toward the next generation of farmers, and the transfer of rights and responsibilities, inter-generational differences among men within households may be as important to take into consideration as differences between men and women.

## Figures and Tables

**Table 6:** Comparison of means for explanatory variables, by plot management

Variables	Male-managed	Female-managed	*p*-Value
	Mean (SD)		
Age	37.5 (17.1)	39.8 (14.5)	0.000
Education	0.23 (0.42)	0.046 (0.21)	0.000
Head	0.38 (0.48)	0.03 (0.17)	0.000
Married	0.68 (0.46)	0.83 (0.37)	0.000
Credit	0.03 (0.17)	0.018 (0.13)	0.000
Extension	4.89 (0.55)	4.94 (0.44)	0.000
Location	0.58 (0.49)	0.61 (0.49)	0.003
Lowland	0.083 (0.28)	0.067 (0.25)	0.006
Slope	0.070 (0.26)	0.063 (0.24)	0.145
Size	0.46 (0.61)	0.29 (0.30)	0.000
Children/female ratio	2.40 (1.32)	2.45 (1.26)	0.047
Female adults	3.08 (2.08)	3.42 (2.07)	0.000
Male adults	2.65 (1.66)	2.03 (1.36)	0.000
Landholding^∗^	3.85 (3.38)	3.93 (3.11)	0.109
Livestock	1.71 (0.91)	1.68 (0.82)	0.053
Non-farm income^∗^	7.45 (5.70)	7.37 (5.67)	0.248
Cotton hectares^∗^	0.24 (1.13)	0.25 (1.01)	0.328
Tenure	0.53 (0.43)	0.50 (0.39)	0.002
Association	0.10 (0.16)	0.11 (0.17)	0.036
Number of agro-dealers	0.54 (0.65)	0.54 (0.64)	0.299
Population density	81.9 (120.6)	92.0 (115.4)	0.000
Rainfall	0.09 (0.04)	0.08 (0.04)	0.000
Millet	0.24 (0.43)	0.35 (0.48)	0.000
Maize	0.25 (0.43)	0.07 (0.25)	0.000

*Source:* As prepared by authors. ∗ denotes time-variant household variables.

**Table 7:** Univariate probit model results, with marginal effects—all individual plots

Independent variables	Yield-enhancing (Set 1)	Marginal effects	Yield-protecting (Set 2)	Marginal effects	Soil-restoring (Set 3)	Marginal effects
*Plot manager characteristics*						
Female	−0.143^∗∗^	−0.0221^∗^	0.0114	0.00258	−0.190^∗∗∗^	−0.0571^∗∗∗^
	(0.0726)	(0.0115)	(0.0613)	(0.0139)	(0.0551)	(0.0169)
Age	−0.00208	−0.000314	−0.00314^∗^	−0.000712^∗^	0.00727^∗∗∗^	0.00214^∗∗∗^
	(0.00192)	(0.000289)	(0.00165)	(0.000374)	(0.00150)	(0.000440)
Education	0.109	0.0171	−0.0232	−0.00521	−0.0397	−0.0116
	(0.0861)	(0.0142)	(0.0784)	(0.0175)	(0.0660)	(0.0190)
Head	−0.197^∗∗^	−0.0276^∗∗^	−0.0465	−0.0104	0.0126	0.00373
	(0.0942)	(0.0123)	(0.0889)	(0.0195)	(0.0702)	(0.0208)
Married	0.276^∗∗∗^	0.0378^∗∗∗^	0.105	0.0231^∗^	0.121^∗∗^	0.0347^∗∗^
	(0.0698)	(0.00877)	(0.0642)	(0.0136)	(0.0551)	(0.0154)
Credit	0.400^∗∗∗^	0.0732^∗∗^	0.191	0.0469	−0.120	−0.0341
	(0.146)	(0.0319)	(0.140)	(0.0368)	(0.132)	(0.0359)
Extension	−0.0560	−0.00843	−0.0990^∗∗^	−0.0224^∗∗^	0.00193	0.000569
	(0.0456)	(0.00687)	(0.0476)	(0.0108)	(0.0411)	(0.0121)

*Plot characteristics*						
Location	0.105^∗^	0.0156^∗^	0.134^∗∗^	0.0300^∗∗^	−0.212^∗∗∗^	−0.0636^∗∗∗^
	(0.0601)	(0.00879)	(0.0561)	(0.0123)	(0.0479)	(0.0145)
Lowland (bas−fond)	−0.0120	−0.00180	−0.0542	−0.0120	−0.0510	−0.0148
	(0.0982)	(0.0146)	(0.0819)	(0.0178)	(0.0780)	(0.0223)
Slope (versant)	0.194^∗∗^	0.0319^∗∗^	−0.0611	−0.0135	0.243^∗∗∗^	0.0758^∗∗∗^
	(0.0837)	(0.0148)	(0.0845)	(0.0183)	(0.0726)	(0.0238)
Size	0.256^∗∗∗^	0.0385^∗∗∗^	0.184^∗∗∗^	0.0417^∗∗∗^	0.0706	0.0208
	(0.0546)	(0.00820)	(0.0505)	(0.0114)	(0.0449)	(0.0132)

*Household characteristics*						
Children/female ratio	0.0280	0.00421	0.0244	0.00554	0.0516^∗∗∗^	0.0152^∗∗∗^
	(0.0254)	(0.00382)	(0.0234)	(0.00528)	(0.0193)	(0.00568)
Female adults	0.0621^∗∗∗^	0.00934^∗∗∗^	−0.0256	−0.00579	0.0402^∗^	0.0118^∗^
	(0.0203)	(0.00307)	(0.0254)	(0.00572)	(0.0207)	(0.00612)
Male adults	0.0278	0.00418	0.0551^∗∗^	0.0125^∗∗^	−0.00481	−0.00142
	(0.0357)	(0.00538)	(0.0265)	(0.00599)	(0.0260)	(0.00764)
Landholding	−0.0207	−0.00311	0.00554	0.00125	−0.0540^∗∗∗^	−0.0159^∗∗∗^
	(0.0202)	(0.00304)	(0.0212)	(0.00481)	(0.0197)	(0.00576)
Livestock	0.00385	0.000580	0.155^∗∗∗^	0.0352^∗∗∗^	0.128^∗∗∗^	0.0377^∗∗∗^
	(0.0442)	(0.00665)	(0.0417)	(0.00949)	(0.0360)	(0.0105)
Non-farm income	0.00629	0.000946	−0.00580	−0.00131	−0.0105^∗^	−0.00310^∗^
	(0.00690)	(0.00104)	(0.00685)	(0.00155)	(0.00572)	(0.00169)
Cotton hectares	0.130^∗∗^	0.0196^∗∗^	−0.0533	−0.0121	0.212^∗∗^	0.0623^∗∗^
	(0.0605)	(0.00912)	(0.0458)	(0.0104)	(0.0851)	(0.0251)
Tenure	0.111	0.0167	−0.000950	−0.000215	0.207^∗∗∗^	0.0610^∗∗∗^
	(0.0706)	(0.0107)	(0.0711)	(0.0161)	(0.0570)	(0.0166)

*Market-related characteristics*						
Association	0.775^∗∗∗^	0.117^∗∗∗^	0.953^∗∗∗^	0.216^∗∗∗^	0.0388	0.0114
	(0.203)	(0.0314)	(0.159)	(0.0365)	(0.172)	(0.0507)
Number of agro-dealers	−0.00599^∗∗∗^	−0.000901^∗∗∗^	0.00490^∗∗∗^	0.00111^∗∗∗^	−0.00746^∗∗∗^	−0.00219^∗∗∗^
	(0.00202)	(0.000310)	(0.00149)	(0.000334)	(0.00169)	(0.000495)
Population density	0.000311	4.69e−05	−0.00314^∗∗^	−0.000712^∗∗^	0.000718^∗∗^	0.000211^∗∗^
	(0.000358)	(5.40e−05)	(0.00130)	(0.000291)	(0.000297)	(8.76e−05)

*Agro-climatic characteristics*						
Rainfall	5.135^∗∗∗^	0.773^∗∗∗^	−1.338^∗^	−0.303^∗^	4.414^∗∗∗^	1.298^∗∗∗^
	(0.709)	(0.113)	(0.753)	(0.172)	(0.650)	(0.191)
Millet	−0.0690	−0.0103	−0.302^∗∗∗^	−0.0650^∗∗∗^	−0.130^∗∗∗^	−0.0378^∗∗∗^
	(0.0656)	(0.00961)	(0.0599)	(0.0123)	(0.0465)	(0.0134)
Maize	1.213^∗∗∗^	0.289^∗∗∗^	0.0336	0.00770	0.339^∗∗∗^	0.108^∗∗∗^
	(0.0727)	(0.0221)	(0.0711)	(0.0164)	(0.0661)	(0.0222)

Number of observations	9,050		9,050		9,050	

∗∗∗, ∗∗ and ∗ denote significance at 1%, 5% and 10%. All regressions include time dummy and household time-average variables. Robust clustered standard errors at the household level.

**Table 1:** Definitions and summary statistics of the intensification strategy sets

Strategies	Definitions	All individually managed	Male-managed	Female-managed	*p*-Value
		Percentage			
Yield-enhancing	Inorganic fertilizer (Urea and/or NPK) and/or improved variety of seed is applied to the plot on which the crop is grown	10.7	15.2	8.2	0.000
Yield-protecting	Fungicide, herbicide, and/or pesticide (liquid or solid) is applied to the plot on which the crop is grown	16.7	18.6	15.7	0.000
Soil-restoring	Manure, compost pit, household refuse, other penning, planting pits (zaï), half-moons (demi-lunes), and/or grass bands (bandes enherbées), are used on plot on which the crop is grown	25.0	29.6	22.6	0.000

*Source:* As prepared by authors. Total *n* = 9,659 individually managed cereal plots, male-managed *n* = 3,369, female-managed *n* = 6,290.

**Table 2:** Definitions and summary statistics of the explanatory variables

Variables	Definition	All individual plots
		Mean (SD)
*Plot manager characteristics*		
Female	If the plot manager is female = 1; Otherwise = 0	0.65
		(0.48)
Age	Age of the plot manager (years)	39.0
		(15.4)
Education	If the plot manager has a primary education = 1; Otherwise = 0	0.11
		(0.31)
Head	If the plot manager is the household head = 1; Otherwise = 0	0.15
		(0.36)
Married	If the plot manager is married = 1; Otherwise = 0	0.78
		(0.41)
Credit	If the plot manager has had access to credit over the last 12 months = 1; Otherwise = 0	0.022
		(0.15)
Extension	Number of years since the plot manager has received any extension services (years). Top-coded at 5 years	4.92
		(0.48)

*Plot characteristics*		
Location	If the plot is located outside the household compound = 1; Otherwise = 0	0.60
		(0.49)
Lowland	If it is a lowland plot = 1 Otherwise = 0	0.072
		(0.26)
Slope	If it is a plot with a steep slope = 1; Otherwise = 0	0.065
		(0.25)
Size	Size of the plot (hectares)	0.35
		(0.46)

*Household characteristics*		
Children/female ratio	Ratio of children to females at the household level (persons)	2.43
		(1.28)
Female adults	Number of female adults in the household (persons)	3.30
		(2.08)
Male adults	Number of male adults in the household (persons)	2.25
		(1.50)
Landholding^∗^	Total land cultivated by the household (hectares)	3.90
		(3.21)
Livestock	Number of livestock owned by the household- measured in tropical livestock units (ln TLU)	1.69
		(0.85)
Non-farm income^∗^	Value of non-farm income at the household level (ln CFA)	7.40
		(5.68)
Cotton hectares^∗^	Number of cotton hectares cultivated at the household level (hectares)	0.25
		(1.05)
Tenure	Share of plots with secure rights (customary landholder or modern form of lease agreement, land certificate, or farming permit) over total plots at the household level	0.51
		(0.40)

*Market-related characteristics*		
Association	Share of households per village belonging to a cereal association	0.104
		(0.166)
Number of agro-dealers	Number of agro-dealers in each province (units)	24.0
		(23.3)
Population density	Number of inhabitants per 100 km^2^ in each province (units)	88.4
		(117.3)

*Agro-climatic characteristics*		
Rainfall	Coefficient of variation of rainfall in each commune over the last three years (mm)	0.088
		(0.042)
Millet	If millet is cultivated on the plot = 1; Otherwise = 0	0.31
		(0.46)
Maize	If maize is cultivated on the plot = 1; Otherwise = 0	0.13
		(0.33)

*Source:* As prepared by authors. ∗ denotes time-variant household variables. Total *n* = 9,659 individually managed cereal plots.

**Table 3:** Multivariate probit model results—all individual plots

Independent variables	Yield-enhancing	Yield-protecting	Soil-restoring
*Plot manager characteristics*			
Female	−0.144^∗∗^	0.0202	−0.187^∗∗∗^
	(0.0718)	(0.0613)	(0.0550)
Age	−0.00227	−0.00337^∗∗^	0.00720^∗∗∗^
	(0.00191)	(0.00165)	(0.00150)
Education	0.0979	−0.0219	−0.0382
	(0.0864)	(0.0787)	(0.0660)
Head	−0.191^∗∗^	−0.0277	0.0136
	(0.0933)	(0.0886)	(0.0699)
Married	0.270^∗∗∗^	0.101	0.119^∗∗^
	(0.0688)	(0.0640)	(0.0550)
Credit	0.391^∗∗∗^	0.207	−0.122
	(0.145)	(0.139)	(0.132)
Extension	−0.0578	−0.0963^∗∗^	0.00233
	(0.0451)	(0.0473)	(0.0413)

*Plot characteristics*			
Location	0.110^∗^	0.140^∗∗^	−0.209^∗∗∗^
	(0.0593)	(0.0560)	(0.0481)
Lowland (bas-fond)	0.00805	−0.0518	−0.0460
	(0.0962)	(0.0814)	(0.0777)
Slope (versant)	0.199^∗∗^	−0.0529	0.244^∗∗∗^
	(0.0833)	(0.0844)	(0.0729)
Size	0.257^∗∗∗^	0.184^∗∗∗^	0.0730
	(0.0551)	(0.0501)	(0.0450)

*Household characteristics*			
Children/female ratio	0.0253	0.0218	0.0509^∗∗∗^
	(0.0248)	(0.0234)	(0.0194)
Female adults	0.0588^∗∗∗^	−0.0267	0.0396^∗^
	(0.0199)	(0.0256)	(0.0208)
Male adults	0.0261	0.0554^∗∗^	−0.00628
	(0.0352)	(0.0261)	(0.0260)
Landholding	−0.0194	0.00706	−0.0537^∗∗∗^
	(0.0202)	(0.0212)	(0.0196)
Livestock	0.0101	0.156^∗∗∗^	0.129^∗∗∗^
	(0.0433)	(0.0418)	(0.0359)
Non-farm income	0.00552	−0.00570	−0.0104^∗^
	(0.00682)	(0.00686)	(0.00572)
Cotton hectares	0.130^∗∗^	−0.0526	0.216^∗∗^
	(0.0598)	(0.0453)	(0.0842)
Tenure	0.103	−0.00295	0.202^∗∗∗^
	(0.0690)	(0.0706)	(0.0567)

*Market characteristics*			
Association	0.733^∗∗∗^	0.934^∗∗∗^	0.0182
	(0.201)	(0.157)	(0.174)
Number of agro-dealers	−0.00554^∗∗∗^	0.00472^∗∗∗^	−0.00727^∗∗∗^
	(0.00192)	(0.00147)	(0.00165)
Population density	0.000278	−0.00326^∗∗^	0.000692^∗∗^
	(0.000353)	(0.00141)	(0.000292)

*Agro-climatic characteristics*			
Rainfall	4.922^∗∗∗^	−1.357^∗^	4.393^∗∗∗^
	(0.708)	(0.756)	(0.651)
Millet	−0.0711	−0.308^∗∗∗^	−0.130^∗∗∗^
	(0.0645)	(0.0599)	(0.0466)
Maize	1.207^∗∗∗^	0.0146	0.351^∗∗∗^
	(0.0726)	(0.0718)	(0.0655)

	Coefficient	Std. error	

rho21	0.309^∗∗∗^	(0.0372)	
rho31	0.190^∗∗∗^	(0.0292)	
rho32	0.0911^∗∗∗^	(0.0286)	

Likelihood ratio test rho21 = rho31 = rho32: chi2(3) = 236.778, Prob > chi2 = 0.0000			
Number of observations: 9,050			

∗∗∗, ∗∗ and ∗ denote significance at 1%, 5% and 10%. All regressions include time dummy and household time-average variables. Robust clustered standard errors at the household level.

**Table 4:** Multivariate probit model results-female managed plots

Independent variables	Yield-enhancing	Yield-protecting	Soil-restoring
*Plot manager characteristics*			
Age	−0.00244	−0.00281	0.00562^∗∗∗^
	(0.00244)	(0.00190)	(0.00183)
Education	−0.0604	0.0922	−0.0603
	(0.184)	(0.131)	(0.103)
Head	−0.0549	0.107	−0.153
	(0.245)	(0.206)	(0.178)
Married	0.275^∗∗∗^	0.0520	0.0601
	(0.103)	(0.0857)	(0.0771)
Credit	0.353^∗^	0.324^∗^	0.0465
	(0.200)	(0.180)	(0.160)
Extension	−0.0442	−0.00496	0.0354
	(0.0638)	(0.0577)	(0.0645)

*Plot characteristics*			
Location	0.00579	0.117^∗^	−0.172^∗∗∗^
	(0.0739)	(0.0639)	(0.0579)
Lowland (bas-fond)	0.255^∗∗^	−0.0184	0.00475
	(0.114)	(0.105)	(0.100)
Slope (versant)	0.251^∗∗^	−0.0955	0.338^∗∗∗^
	(0.108)	(0.0995)	(0.0910)
Size	0.235^∗∗^	0.191^∗∗^	−0.0686
	(0.0938)	(0.0895)	(0.0940)

*Household characteristics*			
Children/female ratio	0.0505	0.0180	0.0611^∗∗^
	(0.0329)	(0.0292)	(0.0256)
Female adults	0.0484^∗^	−0.0462	0.0544^∗∗^
	(0.0271)	(0.0297)	(0.0258)
Male adults	0.0213	0.0904^∗∗∗^	−0.000350
	(0.0473)	(0.0334)	(0.0310)
Landholding	−0.0261	−0.0220	−0.0533^∗∗^
	(0.0317)	(0.0245)	(0.0268)
Livestock	0.0540	0.156^∗∗∗^	0.0701
	(0.0573)	(0.0564)	(0.0451)
Non-farm income	0.00110	−0.00522	−0.00639
	(0.00898)	(0.00823)	(0.00701)
Cotton hectares	0.0882	−0.00122	0.0436
	(0.0649)	(0.0496)	(0.137)
Tenure	0.184^∗∗^	0.0977	0.267^∗∗∗^
	(0.0862)	(0.0881)	(0.0738)

*Market characteristics*			
Association	0.915^∗∗∗^	0.909^∗∗∗^	0.477^∗∗^
	(0.218)	(0.183)	(0.195)
Number of agro-dealers	−0.0119^∗∗∗^	0.00288	−0.00555^∗∗^
	(0.00291)	(0.00193)	(0.00222)
Population density	0.000375	−0.00308^∗^	0.000188
	(0.000524)	(0.00180)	(0.000368)

*Agro-climatic characteristics*			
Rainfall	6.241^∗∗∗^	−1.634^∗^	5.489^∗∗∗^
	(0.867)	(0.923)	(0.795)
Millet	−0.0708	−0.294^∗∗∗^	−0.165^∗∗∗^
	(0.0753)	(0.0690)	(0.0540)
Maize	1.199^∗∗∗^	−0.171	0.318^∗∗∗^
	(0.111)	(0.107)	(0.111)

	Coefficient	Std. error	*p*-Value

rho21	0.263^∗∗∗^	(0.0449)	
rho31	0.230^∗∗∗^	(0.0365)	
rho32	0.190^∗∗∗^	(0.0340)	

Likelihood ratio test rho21 = rho31 = rho32: chi2(3) = 80.9571, Prob > chi2 = 0.0000			
Number of observations: 5,972			

∗∗∗, ∗∗ and ∗ denote significance at 1%, 5% and 10%. All regressions include time dummy and household time-average variables. Robust clustered standard errors at the household level.

**Table 5:** Multivariate probit model results-male managed plots

Independent variables	Yield-enhancing (Set 1)	Yield-protecting (Set 2)	SWC (Set 3)
*Plot manager characteristics*			
Age	−0.00211	−0.00453	0.00860^∗∗∗^
	(0.00319)	(0.00309)	(0.00251)
Education	0.162	−0.0963	0.0302
	(0.102)	(0.0988)	(0.0847)
Head	−0.0699	−0.0465	0.0419
	(0.120)	(0.106)	(0.0918)
Married	0.259^∗∗^	0.152	0.141^∗^
	(0.104)	(0.0942)	(0.0844)
Credit	0.346^∗^	0.0866	−0.351^∗∗^
	(0.183)	(0.202)	(0.177)
Extension	−0.0976^∗^	−0.186^∗∗∗^	−0.0367
	(0.0581)	(0.0655)	(0.0619)

*Plot characteristics*			
Location	0.378^∗∗∗^	0.192^∗∗^	−0.195^∗∗∗^
	(0.0854)	(0.0850)	(0.0650)
Lowland (bas-fond)	−0.326^∗∗^	−0.0661	−0.119
	(0.140)	(0.116)	(0.107)
Slope (versant)	0.178	−0.000383	0.108
	(0.118)	(0.120)	(0.110)
Size	0.231^∗∗∗^	0.172^∗∗∗^	0.120^∗∗^
	(0.0584)	(0.0570)	(0.0495)

*Household characteristics*			
Children/female ratio	−0.0192	0.0338	0.0345
	(0.0313)	(0.0300)	(0.0249)
Female adults	0.0886^∗∗∗^	0.0107	0.0116
	(0.0269)	(0.0340)	(0.0250)
Male adults	0.00739	−0.0125	0.0189
	(0.0467)	(0.0317)	(0.0359)
Landholding	−0.00657	0.0516^∗^	−0.0578^∗∗^
	(0.0255)	(0.0267)	(0.0254)
Livestock	−0.0104	0.158^∗∗∗^	0.200^∗∗∗^
	(0.0510)	(0.0468)	(0.0467)
Non-farm income	0.0125	−0.00379	−0.0175^∗∗^
	(0.00999)	(0.0103)	(0.00778)
Cotton hectares	0.154^∗^	−0.0962	0.251^∗∗^
	(0.0853)	(0.0806)	(0.103)
Tenure	−0.0704	−0.161^∗^	0.116
	(0.0938)	(0.0922)	(0.0763)

*Market characteristics*			
Association	0.175	0.955^∗∗∗^	−0.697^∗∗∗^
	(0.333)	(0.230)	(0.260)
Number of agro-dealers	0.000128	0.00803^∗∗∗^	−0.00733^∗∗∗^
	(0.00213)	(0.00164)	(0.00203)
Population density	0.000141	−0.00383^∗∗^	0.00112^∗∗∗^
	(0.000395)	(0.00188)	(0.000379)

*Agro-climatic characteristics*			
Rainfall	2.258^∗∗^	−1.459	2.234^∗∗∗^
	(1.013)	(0.997)	(0.836)
Millet	−0.0270	−0.333^∗∗∗^	−0.101
	(0.104)	(0.0918)	(0.0755)
Maize	1.292^∗∗∗^	0.144	0.372^∗∗∗^
	(0.0969)	(0.0889)	(0.0748)

	Coefficient	Std. error	*p*-Value

rho21	0.301^∗∗∗^	(0.0500)	
rho31	0.177^∗∗∗^	(0.0432)	
rho32	0.0261	(0.0404)	

Likelihood ratio test rho21 = rho31 = rho32: chi2(3) = 167.948, Prob > chi2 = 0.0000			
Number of observations: 3,078			

∗∗∗, ∗∗ and ∗ denote significance at 1%, 5% and 10%. All regressions include time dummy and household time-average variables. Robust clustered standard errors at the household level.

## Footnotes

1 Compared with univariate probit models, estimating marginal effects and standard errors for multivariate probit models is “extremely messy” (Greene, 2010) and can be computationally infeasible (Hsiao, 2014, p. 235). Our attempt to compute them failed after a week of iterations.
